# *In vitro *calibration of a system for measurement of *in vivo *convective heat transfer coefficient in animals

**DOI:** 10.1186/1475-925X-5-57

**Published:** 2006-10-26

**Authors:** Chanchana Tangwongsan, Louay Chachati, John G Webster, Patrick V Farrell

**Affiliations:** 1 Department of Electrical Engineering, Chulalongkorn University Phaya-Thai Road, Bangkok 10330, Thailand; 2 Department of Electrical and Electronic Engineering, University of Aleppo, Aleppo, Syria; 3 Department of Biomedical Engineering, University of Wisconsin, 1550 Engineering Drive, Madison, WI 53706, USA; 4 Department of Mechanical Engineering, University of Wisconsin, Madison, WI 53706, USA

## Abstract

**Background:**

We need a sensor to measure the convective heat transfer coefficient during ablation of the heart or liver.

**Methods:**

We built a minimally invasive instrument to measure the *in vivo *convective heat transfer coefficient, *h *in animals, using a Wheatstone-bridge circuit, similar to a hot-wire anemometer circuit. One arm is connected to a steerable catheter sensor whose tip is a 1.9 mm × 3.2 mm thin film resistive temperature detector (RTD) sensor. We used a circulation system to simulate different flow rates at 39°C for *in vitro *experiments using distilled water, tap water and saline. We heated the sensor approximately 5°C above the fluid temperature. We measured the power consumed by the sensor and the resistance of the sensor during the experiments and analyzed these data to determine the value of the convective heat transfer coefficient at various flow rates.

**Results:**

From 0 to 5 L/min, experimental values of *h *in W/(m^2^·K) were for distilled water 5100 to 13000, for tap water 5500 to 12300, and for saline 5400 to 13600. Theoretical values were 1900 to 10700.

**Conclusion:**

We believe this system is the smallest, most accurate method of minimally invasive measurement of *in vivo h *in animals and provides the least disturbance of flow.

## Background

Radiofrequency (RF) catheter ablation has been used to treat many types of cardiac arrhythmias such as atrial tachyarrhythmias (atrial tachycardia, atrial flutter, atrial fibrillation), atrioventricular nodal re-entrant tachycardia, Wolf-Parkinson-White syndrome, symptomatic supraventricular and ventricular tachycardia (fascicular VT, bundle branch re-entrant VT, idiopathic VT, ischemic VT) [1-5] with high success rate because of its controllability, high efficacy, low complications and minimal invasiveness. The technique of radiofrequency catheter ablation is to deliver high-frequency alternating electric current from 350 kHz to 1 MHz through the electrode catheter to generate a thermal lesion in myocardial tissue [6]. The tissue in direct contact with the catheter is heated by resistive (ohmic) heating. Resistive heating in tissue is proportional to the power density [6]. The thermal energy from the directly contacted tissue is then transferred to its vicinity by means of conduction and forms an RF lesion. Flowing blood near the tissue transfers heat away by convection and is a major cause of heat loss from the RF lesion. Convective heat transferred by the epicardial coronary artery is considered a minor heat loss in RF catheter ablation [6,7].

Hot wire/film anemometer systems have been used to measure the blood velocity *in vivo*. Nerem et al. [8] used a hot film probe to study the velocity distribution in the aortas of dogs. Paulsen and Nissen [9] developed a safety system for a hot-film anemometer for blood-velocity measurement in humans. Yamaguchi et al. [10] performed turbulence measurements in the center of the canine ascending aorta using a hot-film anemometer. Paulsen et al. [11] analyzed the dynamic properties of a hot-film anemometer system for blood velocity measurements in humans.

In order to improve the electrodes and success rate of RF catheter ablation, researchers have used finite element method (FEM) modelling to simulate the ablation [7,12,13]. The value of the endocardial convective heat transfer coefficient (*h*) is essential for the simulation of heat loss from the endocardium to the blood pool in the cardiac chambers [7,13]. Researchers have used values of *h *ranging from 44 to 6090 W/(m^2^·K) for various locations in the cardiac chambers [7,13-15], however, none of those values came from *in vivo *measurement in animals. Because we did not find *in vivo *measurements of *h *in animals, we built our instrument, which is a Wheatstone-bridge circuit connected to a thin film resistive temperature detector (RTD) sensor, similar to a hot-wire anemometer circuit, and used it to measure the endocardial *h *first *in vitro *and then *in vivo *[16].

Absolute accuracy in measuring heat transfer coefficient *h *might be advantageous, but will be very difficult to attain given the real geometry of the sensor and the operating conditions envisioned for its use. Of equal value, and far more realizable, is a sensor that can provide repeatable, reasonably accurate estimates of local *h*, exhibiting the variations in time and space expected in the *in vivo *application. We will demonstrate the sensor described provides repeatable results in *in vitro *experiments, which are consistent with an estimated lower bound on *h *from heat transfer correlations on an idealized geometry.

## Methods

**Figure 1:**
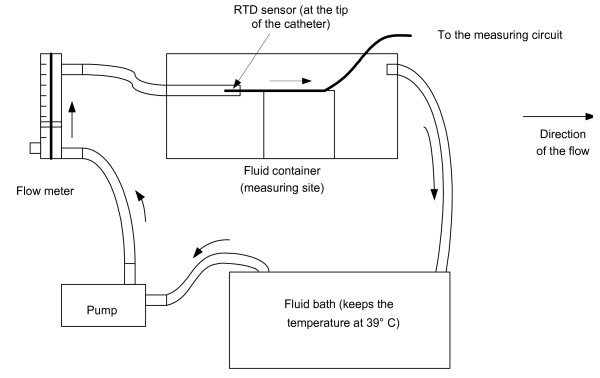
The circulation system consists of a pump connected to a flow meter, a container, which is the measuring site for the RTD sensor probe, and a fluid bath, which maintains the temperature of the fluid at 39°C

**Figure 2:**
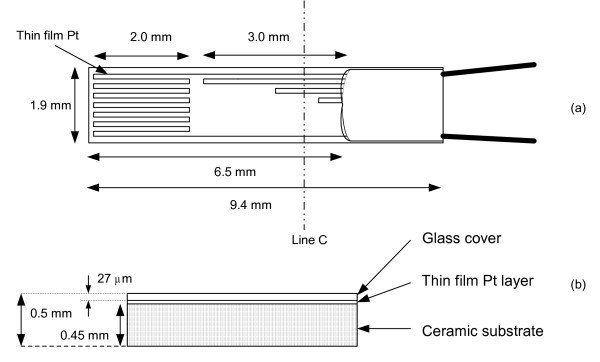
The RTD sensor has a thin film Pt layer overcoated with a glass layer and a ceramic substrate, (a) Top view of the RTD sensor, showing the line pattern of the thin film Pt, and (b) Cross-sectional view of the RTD sensor through line C, showing the thickness of the glass layer of 27 μm and the whole thickness of the sensor of 0.5 mm.

**Figure 3:**
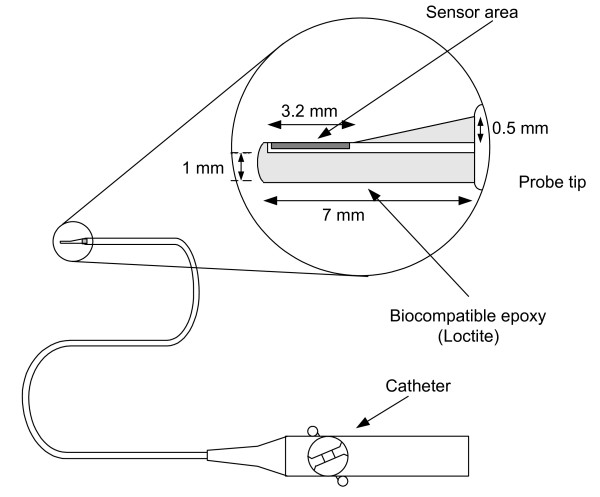
The Pt sensor is placed at the tip of a catheter probe surrounded by epoxy, leaving the exposed area of 1.9 × 3.2 mm. The turnable knob on the catheter handle controls catheter tip movement.

**Figure 4:**
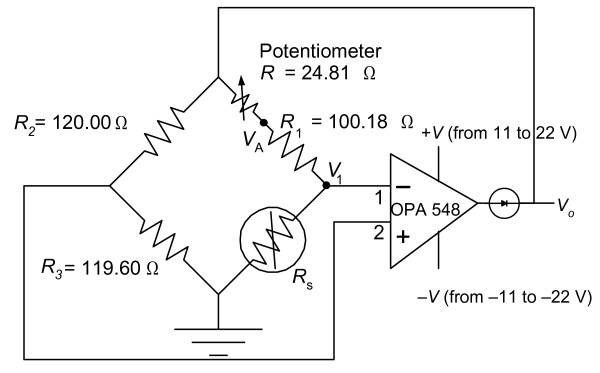
Circuit diagram of constant temperature measuring system, *R*_1_, *R*_2_, and *R*_3 _are wire-wound resistors with 1% tolerance, ±20 ppm/°C temperature coefficient. *R*_s _is a resistive temperature detector (RTD).

In order to obtain the value of *h *inside the cardiac chambers, we performed *in vitro *experiments to ensure the capability of our measuring system, to test for any leakage current and to calibrate the system. The *in vitro *experiment setting was consisted of a circulation system (Fig. 1), the sensor (Figs. 2 and 3), the measuring circuit (Fig. 4) and the data acquisition program. The circulation system simulated flow rates of blood from 0 to 5 L/min to provide different flow rates for three different fluids (distilled water, tap water and saline). The catheter sensor, whose tip is a Pt thin film resistive temperature detector (RTD) sensor, was placed in the fluid flow, and formed one arm of the Wheatstone bridge circuit (the measuring circuit). The electric power consumed by the sensor and the temperature of the sensor were measured and saved using a data acquisition program and were then analyzed to yield *h *(using equation (16)).

### A. Circulation system

Fig. 1 shows that the circulation system was composed of a fluid bath, a centrifugal pump, a flow meter and a fluid container. We measured flow with a rotameter Model 7200–0061 (King Instrument Co., Garden Grove, CA), which has a full scale of 2 GPM (8 L/min) and 3% accuracy. The fluid bath maintained the temperature of the fluid at 39°C (swine body temperature). From the fluid bath, the pump pumped the fluid to the flow meter. A valve at the flow meter was adjusted to vary the flow rate from 0 to 5 L/min. The fluid then flowed to the container through a 20 mm diameter, 500 mm long, solid PVC tube with the heated sensor on the axis in the tube.

Since the tube was more than 20 times longer than the diameter of the tube, this suggests that the expected laminar flow was fully developed at the measuring point. As the fluid flowed past the sensor, it dissipated heat from the sensor and cooled the sensor by means of convection. The higher the flow rate, the faster the heat was dissipated.

Because this system will be used to measure the endocardial convective heat transfer coefficient, *h*, *in vivo*, it is important that the measuring system (especially, the catheter sensor) will not create any leakage current while performing the *h *measurements. Since the electrical conductivity (σ) of blood is around 0.60 to 0.67 S/m at 20 to 25°C [17], we must perform the *in vitro *tests in fluid that has electrical conductivity similar to that of blood. Therefore, three different types of fluid were used: distilled water, tap water and saline. Distilled water (σ ≈ 10^-4 ^to 10^-3 ^S/m [18]), which has a very low electrical conductivity, was first used for the *in vitro *experiments (as control experiments) in order to avoid having any leakage current during the experiment. Tap water (σ ≈ 0.15 S/m [19]), which has an electrical conductivity higher than that of distilled water but lower than that of blood, and saline (σ ≈ 0.82 S/m [20]), which has electrical conductivity slightly higher than that of blood, were later used in the experiments in order to determine if any leakage current was created from the catheter sensor as well as to observe the differences of *h *obtained from these fluids with different electrical conductivity.

### B. Measuring circuit

Fig. 4 shows our measuring system (similar to a hot wire/film anemometer system) using a resistive temperature detector (RTD) (Fig. 2), *R*_s_, forming one arm of a Wheatstone bridge. This circuit maintained the resistance of the sensor (*R*_s_) at a constant value, hence holding the temperature of the sensor constant (constant temperature mode). During the measurement, the sensor was heated approximately 5°C above the temperature of the flowing fluid (distilled water, tap water and saline). The electric power consumed by the sensor and the resistance of the sensor during the experiment were recorded and analyzed to yield the value of *h*.

The Wheatstone bridge had a resistive temperature detector (RTD) sensor in one arm of the bridge, and three wire-wound resistors (1 W), and a precision potentiometer in the other arms. A power operational amplifier supplied the power to the circuit and the sensor. The resistance of the sensor was maintained constant by the bridge at *R*_S _= *R*_R _(*G *- 2)/(*G *+ 2) where *R*_R _is the total resistance of the upper right arm of the bridge, and *G *is the gain of the op amp [21]. The sensor was electrically heated about 5°C above the fluid temperature because if using this system *in vivo*, higher temperatures may cause blood coagulation on the surface of the sensor and may damage the surrounding cells [6]. Therefore we restricted the temperature difference to 5°C also for the *in vitro *tests. Dissipation of the heat occurred because the flowing fluid carried the warmed fluid away from the surface of the heated sensor. Increased velocity of the fluid reduced the fluid temperature next to the sensor and therefore the ohmic resistance of the sensor. Restoration of the sensor to its original working temperature was achieved by feedback controlled by the op amp. The sensor current increased to increase the power and the temperature of the sensor.

The voltage across the sensor (*V*_1_) and the voltage across *R*_1 _and *R*_S _(*V*_A_) (see Fig. 4) were recorded by the data acquisition program through the analog-to-digital converter (ADC) (12 bit, 100 kS/s, 8 analog inputs). *V*_A _and *V*_1 _were used to determine the current that flowed through the sensor. *V*_1 _was also used to determine the resistance and the temperature of the sensor. Once we knew the value of the current, the resistance and the temperature of the sensor, we calculated *h *using Newton's law of cooling [22]:

*q *= *hA*(*T*_s _- *T*_∞_) = *Q*_h _    (1)

where: *q *is the heat flow (W), *Q*_h_ is the electric power consumed by heating the sensor (W), *h *is the convective heat transfer coefficient (W/(m^2^·K)), *A *is the sensor area (m^2^), *T*_S _is the temperature of the heated sensor (K), and *T*_∞ _is the temperature of the bulk fluid (K). However, since the sensor we used was not a bare Pt thin film, we calculated a correction.

### C. Catheter sensor design

To measure *in vivo h*, we required a catheter that could be externally steered and placed against cardiac chamber and vessel walls. The sensor (model TFD, Omega Company) was at the tip of a cardiac ablation catheter and Loctite sealed the electric connection, covered the backside of the probe and rounded the sharp edges of the sensor. Fig. 2 shows the structure of the thin film Pt sensor. Its size was 1.9 × 9.4mm with a sensing area of 1.9 × 3.2mm (after covering the less-temperature sensitive area and the rough edges with Loctite). Fig. 3 shows the structure of the catheter with the sensor at the tip.

### D. Temperature vs. resistance for the sensor

**Figure 5:**
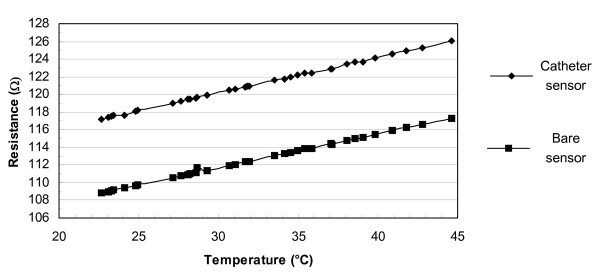
Temperature vs. resistance of the bare sensor and the catheter sensor with the catheter wire immersed in the 37°C

When electrically connecting the bare sensor to a catheter, the overall resistance of the catheter sensor was greater than that of the bare sensor because of the added resistance of the lead wire. We used an adjustable temperature water bath and a digital multimeter (model# HP34401A) to plot the resistance versus the temperature shown in Fig. 5. The average resistance difference between the bare sensor and the sensor with the lead wire was about 8.51 Ω. The resistance differences of the lead wire in air (25°C, room temperature) and in heated water (up to 45°C) were less than 0.1 Ω. Using this information, we used the DIN EN 60751 [23], resistance vs. temperature table. The resistance of the catheter sensor was *R*_c _= *R*_s _+ 8.51 Ω, where *R*_s _was the resistance of the bare sensor.

## Results

### Theoretical calculation of forced convection at the center of a 20 mm diameter tube

It was important to consider the closest theoretical prediction possible in order to guide our experimental testing. Therefore, we calculated the value of forced *h *for laminar flow, constant heat flux [22], using:

Nu=hLk     (2)
 MathType@MTEF@5@5@+=feaafiart1ev1aaatCvAUfKttLearuWrP9MDH5MBPbIqV92AaeXatLxBI9gBaebbnrfifHhDYfgasaacH8akY=wiFfYdH8Gipec8Eeeu0xXdbba9frFj0=OqFfea0dXdd9vqai=hGuQ8kuc9pgc9s8qqaq=dirpe0xb9q8qiLsFr0=vr0=vr0dc8meaabaqaciaacaGaaeqabaqabeGadaaakeaacqqGobGtcqqG1bqDcqGH9aqpdaWcaaqaaiabdIgaOjabdYeambqaaiabdUgaRbaacaWLjaGaaCzcamaabmaabaGaeGOmaidacaGLOaGaayzkaaaaaa@37EE@

where: Nu is the Nusselt number (dimensionless), *k *is thermal conductivity (W/(m·K)), *L *is the length of the heated area (parallel to the direction of the flow), (For our catheter sensor, the length of the exposed, heated area was 3.2 × 10^-3 ^m).

The average Nusselt number (Nu) for laminar flow, constant heat flux from a flat plate heated from *x *= 0 to *x *= *L*, and Re < 5 × 10^5 ^can be estimated from the following equation [22]:

Nu¯
 MathType@MTEF@5@5@+=feaafiart1ev1aaatCvAUfKttLearuWrP9MDH5MBPbIqV92AaeXatLxBI9gBaebbnrfifHhDYfgasaacH8akY=wiFfYdH8Gipec8Eeeu0xXdbba9frFj0=OqFfea0dXdd9vqai=hGuQ8kuc9pgc9s8qqaq=dirpe0xb9q8qiLsFr0=vr0=vr0dc8meaabaqaciaacaGaaeqabaqabeGadaaakeaadaqdaaqaaiabb6eaojabbwha1baaaaa@2F51@ = 0.664(Re^1/2^)(Pr^1/3^)     (3)

Re⁡=ρuLμ     (4)
 MathType@MTEF@5@5@+=feaafiart1ev1aaatCvAUfKttLearuWrP9MDH5MBPbIqV92AaeXatLxBI9gBaebbnrfifHhDYfgasaacH8akY=wiFfYdH8Gipec8Eeeu0xXdbba9frFj0=OqFfea0dXdd9vqai=hGuQ8kuc9pgc9s8qqaq=dirpe0xb9q8qiLsFr0=vr0=vr0dc8meaabaqaciaacaGaaeqabaqabeGadaaakeaacyGGsbGucqGGLbqzcqGH9aqpdaWcaaqaaGGaciab=f8aYjabdwha1jabdYeambqaaiab=X7aTbaacaWLjaGaaCzcamaabmaabaGaeGinaqdacaGLOaGaayzkaaaaaa@3A11@

Pr⁡=CPμk     (5)
 MathType@MTEF@5@5@+=feaafiart1ev1aaatCvAUfKttLearuWrP9MDH5MBPbIqV92AaeXatLxBI9gBaebbnrfifHhDYfgasaacH8akY=wiFfYdH8Gipec8Eeeu0xXdbba9frFj0=OqFfea0dXdd9vqai=hGuQ8kuc9pgc9s8qqaq=dirpe0xb9q8qiLsFr0=vr0=vr0dc8meaabaqaciaacaGaaeqabaqabeGadaaakeaacyGGqbaucqGGYbGCcqGH9aqpdaWcaaqaaiabdoeadnaaBaaaleaacqqGqbauaeqaaGGacOGae8hVd0gabaGaem4AaSgaaiaaxMaacaWLjaWaaeWaaeaacqaI1aqnaiaawIcacaGLPaaaaaa@39A5@

α=(Re⁡St)0.5=D22πfν     (6)
 MathType@MTEF@5@5@+=feaafiart1ev1aaatCvAUfKttLearuWrP9MDH5MBPbIqV92AaeXatLxBI9gBaebbnrfifHhDYfgasaacH8akY=wiFfYdH8Gipec8Eeeu0xXdbba9frFj0=OqFfea0dXdd9vqai=hGuQ8kuc9pgc9s8qqaq=dirpe0xb9q8qiLsFr0=vr0=vr0dc8meaabaqaciaacaGaaeqabaqabeGadaaakeaaiiGacqWFXoqycqGH9aqpdaqadaqaamaalaaabaGagiOuaiLaeiyzaugabaGaee4uamLaeeiDaqhaaaGaayjkaiaawMcaamaaCaaaleqabaGaeGimaaJaeiOla4IaeGynaudaaOGaeyypa0ZaaSaaaeaacqWGebaraeaacqaIYaGmaaWaaOaaaeaadaWcaaqaaiabikdaYiab=b8aWjabdAgaMbqaaiab=17aUbaaaSqabaGccaWLjaGaaCzcamaabmaabaGaeGOnaydacaGLOaGaayzkaaaaaa@45D5@

where: Re is the Reynolds number (dimensionless), Pr is the Prandtl number (dimensionless), *α* is the Womersley number (dimensionless), St is Strouhal number (dimensionless), *ρ* is the density of fluid (kg/m^3^), *μ* is dynamic viscosity (N·s/m^2^), *ν* is the kinematic viscosity = *μ/ρ* (m^2^/s), *C*_P _is specific heat at constant pressure (J/(kg·K)), *u *is the velocity of the flow at the sensor location (m/s)

u=2F(m3/s)At(m2)=2F(L/min⁡)60×1000×At(m2)(m/s)
 MathType@MTEF@5@5@+=feaafiart1ev1aaatCvAUfKttLearuWrP9MDH5MBPbIqV92AaeXatLxBI9gBaebbnrfifHhDYfgasaacH8akY=wiFfYdH8Gipec8Eeeu0xXdbba9frFj0=OqFfea0dXdd9vqai=hGuQ8kuc9pgc9s8qqaq=dirpe0xb9q8qiLsFr0=vr0=vr0dc8meaabaqaciaacaGaaeqabaqabeGadaaakeaacqWG1bqDcqGH9aqpdaWcaaqaaiabikdaYiabdAeagjabcIcaOiabb2gaTnaaCaaaleqabaGaeG4mamdaaOGaei4la8Iaee4CamNaeiykaKcabaGaemyqae0aaSbaaSqaaiabbsha0bqabaGccqGGOaakcqqGTbqBdaahaaWcbeqaaiabikdaYaaakiabcMcaPaaacqGH9aqpdaWcaaqaaiabikdaYiabdAeagjabcIcaOiabbYeamjabc+caViGbc2gaTjabcMgaPjabc6gaUjabcMcaPaqaaiabiAda2iabicdaWiabgEna0kabigdaXiabicdaWiabicdaWiabicdaWiabgEna0kabdgeabnaaBaaaleaacqqG0baDaeqaaOGaeiikaGIaeeyBa02aaWbaaSqabeaacqaIYaGmaaGccqGGPaqkaaGaeiikaGIaeeyBa0Maei4la8Iaee4CamNaeiykaKcaaa@5FD1@

*F *= Flow, *D *is the diameter of the tube (m), *f *is a characteristic frequency of the waveform (in this case, the heart rate) (Hz), *A*_t _is the cross-sectional area of the 20 mm diameter tube = π(0.01)^2 ^= 3.1416 × 10^-4^m^2^.

For the calculation of Nu¯
 MathType@MTEF@5@5@+=feaafiart1ev1aaatCvAUfKttLearuWrP9MDH5MBPbIqV92AaeXatLxBI9gBaebbnrfifHhDYfgasaacH8akY=wiFfYdH8Gipec8Eeeu0xXdbba9frFj0=OqFfea0dXdd9vqai=hGuQ8kuc9pgc9s8qqaq=dirpe0xb9q8qiLsFr0=vr0=vr0dc8meaabaqaciaacaGaaeqabaqabeGadaaakeaadaqdaaqaaiabb6eaojabbwha1baaaaa@2F51@, Re and *h*, the values of Pr, *ρ, μ*, *C*_P_, and *k *at 37°C and 39°C are listed in Table 1. For the *in vitro *experiments, the unsteadiness of the flow was insignificant (with very small value of the Womersley number) and could be neglected. However, in physiological situations, a larger value of the Womersley number would be obtained, thus equations (4) and (5) must be adjusted according to the unsteadiness of the flow due to the heart rate.

**Table 1:** The value of Prandtl number, density, dynamic viscosity, specific heat, and thermal conductivity of water at 37°C and 39°C.

	**At 37°C**	**At 39°C**
Pr	4.6	4.4
*ρ* (kg/m^3^)	993.9	992.47
*μ* (N·s/m^2^)	6.9 × 10^-4^	6.7 × 10^-4^
*C*_P _(J/(kg·K))	4174	4174
*K *(W/(m·K))	0.63	0.63

### Theoretical calculation of free convection for a vertical plane

When placing a heated sensor in a still liquid, heat generated by the sensor is dissipated by free or natural heat convection. The average value of free or natural convection, caused by the fluid movement resulting from the change of the fluid density due to the heating process, can be calculated using [22]:

Nuf¯=C(GrfPr⁡f)m=hxkf     (7)
 MathType@MTEF@5@5@+=feaafiart1ev1aaatCvAUfKttLearuWrP9MDH5MBPbIqV92AaeXatLxBI9gBaebbnrfifHhDYfgasaacH8akY=wiFfYdH8Gipec8Eeeu0xXdbba9frFj0=OqFfea0dXdd9vqai=hGuQ8kuc9pgc9s8qqaq=dirpe0xb9q8qiLsFr0=vr0=vr0dc8meaabaqaciaacaGaaeqabaqabeGadaaakeaadaqdaaqaaiabb6eaojabbwha1naaBaaaleaacqqGMbGzaeqaaaaakiabg2da9iabdoeadjabcIcaOiabbEeahjabbkhaYnaaBaaaleaacqqGMbGzaeqaaOGagiiuaaLaeiOCai3aaSbaaSqaaiabbAgaMbqabaGccqGGPaqkdaahaaWcbeqaaiabd2gaTbaakiabg2da9maalaaabaGaemiAaGMaemiEaGhabaGaem4AaS2aaSbaaSqaaiabbAgaMbqabaaaaOGaaCzcaiaaxMaadaqadaqaaiabiEda3aGaayjkaiaawMcaaaaa@48FC@

where the subscript f indicates that the properties in the dimensionless groups are evaluated at the film temperature (*T*_f_), where:

Tf=T∞−Tw2     (8)
 MathType@MTEF@5@5@+=feaafiart1ev1aaatCvAUfKttLearuWrP9MDH5MBPbIqV92AaeXatLxBI9gBaebbnrfifHhDYfgasaacH8akY=wiFfYdH8Gipec8Eeeu0xXdbba9frFj0=OqFfea0dXdd9vqai=hGuQ8kuc9pgc9s8qqaq=dirpe0xb9q8qiLsFr0=vr0=vr0dc8meaabaqaciaacaGaaeqabaqabeGadaaakeaacqWGubavdaWgaaWcbaGaeeOzaygabeaakiabg2da9maalaaabaGaemivaq1aaSbaaSqaaiabg6HiLcqabaGccqGHsislcqWGubavdaWgaaWcbaGaee4DaChabeaaaOqaaiabikdaYaaacaWLjaGaaCzcamaabmaabaGaeGioaGdacaGLOaGaayzkaaaaaa@3BDA@

where: *T*_∞ _is the fluid temperature (°C), *T*_w _is the wall temperature (of the heated plane) (°C), and for constant heat flux surface:

GrxPr⁡f=(gβρ2CPμk)x3ΔT     (9)
 MathType@MTEF@5@5@+=feaafiart1ev1aaatCvAUfKttLearuWrP9MDH5MBPbIqV92AaeXatLxBI9gBaebbnrfifHhDYfgasaacH8akY=wiFfYdH8Gipec8Eeeu0xXdbba9frFj0=OqFfea0dXdd9vqai=hGuQ8kuc9pgc9s8qqaq=dirpe0xb9q8qiLsFr0=vr0=vr0dc8meaabaqaciaacaGaaeqabaqabeGadaaakeaacqqGhbWrcqqGYbGCdaWgaaWcbaGaeeiEaGhabeaakiGbccfaqjabckhaYnaaBaaaleaacqqGMbGzaeqaaOGaeyypa0ZaaeWaaeaadaWcaaqaaiabdEgaNHGaciab=j7aIjab=f8aYnaaCaaaleqabaGaeGOmaidaaOGaem4qam0aaSbaaSqaaiabbcfaqbqabaaakeaacqWF8oqBcqWGRbWAaaaacaGLOaGaayzkaaGaemiEaG3aaWbaaSqabeaacqaIZaWmaaGccqqHuoarcqWGubavcaWLjaGaaCzcamaabmaabaGaeGyoaKdacaGLOaGaayzkaaaaaa@4BFE@

where: Gr_f _is the Grashof number at the film temperature, Pr_f _is the Prandtl number at the film temperature, *g *is acceleration of gravity (m/s^2^), *β* is the temperature coefficient of thermal conductivity (1/K), *x *is the length of the exposed area of the heated sensor (= 3.2 × 10^-^^3 ^m), Δ*T *is temperature difference of the fluid and the wall (*T*_∞ _- *T*_w_), *C *is a constant, and can be evaluated by the value of Gr_f_Pr_f_, *m *is a constant, and can also be evaluated by the value of Gr_f_Pr_f_, *h *is the local free convective heat transfer coefficient (W/(m^2^·K)).

For free convection from isothermal vertical planes, the values of local *h *and Nu_f _can be analyzed according to Table 2. Since the value of Gr_x_Pr_f _is lower than 10^4^, we used Fig. 7-7 of [22] (free convection heat transfer from vertical isothermal plate, Nu vs. GrPr) to determine the value of Nu¯f
 MathType@MTEF@5@5@+=feaafiart1ev1aaatCvAUfKttLearuWrP9MDH5MBPbIqV92AaeXatLxBI9gBaebbnrfifHhDYfgasaacH8akY=wiFfYdH8Gipec8Eeeu0xXdbba9frFj0=OqFfea0dXdd9vqai=hGuQ8kuc9pgc9s8qqaq=dirpe0xb9q8qiLsFr0=vr0=vr0dc8meaabaqaciaacaGaaeqabaqabeGadaaakeaadaqdaaqaaiabb6eaojabbwha1baadaWgaaWcbaGaeeOzaygabeaaaaa@30D0@ without using the value of *C *and *m*.

**Table 2:** Constants for use with equation (6) for isothermal surface.

**Gr_f_Pr_f_**	*C*	***m***
10^-1 ^– 10^4^	Use Fig. 7-7 of [22]	Use Fig. 7-7 of [22]
10^4 ^– 10^9^	0.59	0.25
10^9 ^– 10^13^	0.10	0.33

The average convective heat transfer coefficient (h¯
 MathType@MTEF@5@5@+=feaafiart1ev1aaatCvAUfKttLearuWrP9MDH5MBPbIqV92AaeXatLxBI9gBaebbnrfifHhDYfgasaacH8akY=wiFfYdH8Gipec8Eeeu0xXdbba9frFj0=OqFfea0dXdd9vqai=hGuQ8kuc9pgc9s8qqaq=dirpe0xb9q8qiLsFr0=vr0=vr0dc8meaabaqaciaacaGaaeqabaqabeGadaaakeaacuWGObaAgaqeaaaa@2E1D@) for laminar region can be evaluated by [22]:

h¯=1L∫0Lhxdx=54hx=L     (10)
MathType@MTEF@5@5@+=feaafiart1ev1aaatCvAUfKttLearuWrP9MDH5MBPbIqV92AaeXatLxBI9gBaebbnrfifHhDYfgasaacH8akY=wiFfYdH8Gipec8Eeeu0xXdbba9frFj0=OqFfea0dXdd9vqai=hGuQ8kuc9pgc9s8qqaq=dirpe0xb9q8qiLsFr0=vr0=vr0dc8meaabaqaciaacaGaaeqabaqabeGadaaakeaacuWGObaAgaqeaiabg2da9maalaaabaGaeGymaedabaGaemitaWeaamaapehabaGaemiAaG2aaSbaaSqaaiabdIha4bqabaGccqWGKbazcqWG4baEaSqaaiabicdaWaqaaiabdYeambqdcqGHRiI8aOGaeyypa0ZaaSaaaeaacqaI1aqnaeaacqaI0aanaaGaemiAaG2aaSbaaSqaaiabdIha4jabg2da9iabdYeambqabaGccaWLjaGaaCzcamaabmaabaGaeGymaeJaeGimaadacaGLOaGaayzkaaaaaa@4876@

In a water bath with constant temperature of 39°C, a sensor was placed vertically. The sensor was heated to a constant 5°C above the water temperature. The free *h *was *h*_local _= 1500 W/(m^2^·K) and h¯
 MathType@MTEF@5@5@+=feaafiart1ev1aaatCvAUfKttLearuWrP9MDH5MBPbIqV92AaeXatLxBI9gBaebbnrfifHhDYfgasaacH8akY=wiFfYdH8Gipec8Eeeu0xXdbba9frFj0=OqFfea0dXdd9vqai=hGuQ8kuc9pgc9s8qqaq=dirpe0xb9q8qiLsFr0=vr0=vr0dc8meaabaqaciaacaGaaeqabaqabeGadaaakeaacuWGObaAgaqeaaaa@2E1D@ = 1870 W/(m^2^·K).

Table 3 shows the values of *u*, Re, Nu and h¯
 MathType@MTEF@5@5@+=feaafiart1ev1aaatCvAUfKttLearuWrP9MDH5MBPbIqV92AaeXatLxBI9gBaebbnrfifHhDYfgasaacH8akY=wiFfYdH8Gipec8Eeeu0xXdbba9frFj0=OqFfea0dXdd9vqai=hGuQ8kuc9pgc9s8qqaq=dirpe0xb9q8qiLsFr0=vr0=vr0dc8meaabaqaciaacaGaaeqabaqabeGadaaakeaacuWGObaAgaqeaaaa@2E1D@. Note that at zero flow rate, h¯
 MathType@MTEF@5@5@+=feaafiart1ev1aaatCvAUfKttLearuWrP9MDH5MBPbIqV92AaeXatLxBI9gBaebbnrfifHhDYfgasaacH8akY=wiFfYdH8Gipec8Eeeu0xXdbba9frFj0=OqFfea0dXdd9vqai=hGuQ8kuc9pgc9s8qqaq=dirpe0xb9q8qiLsFr0=vr0=vr0dc8meaabaqaciaacaGaaeqabaqabeGadaaakeaacuWGObaAgaqeaaaa@2E1D@ is calculated using free convection equations.

**Table 3:** Theoretical value of h¯
 MathType@MTEF@5@5@+=feaafiart1ev1aaatCvAUfKttLearuWrP9MDH5MBPbIqV92AaeXatLxBI9gBaebbnrfifHhDYfgasaacH8akY=wiFfYdH8Gipec8Eeeu0xXdbba9frFj0=OqFfea0dXdd9vqai=hGuQ8kuc9pgc9s8qqaq=dirpe0xb9q8qiLsFr0=vr0=vr0dc8meaabaqaciaacaGaaeqabaqabeGadaaakeaacuWGObaAgaqeaaaa@2E1D@, Re, Nu, and *u *for laminar flow of the catheter sensor in a 20 mm diameter tube.

**Flow (L/min)**	u¯ MathType@MTEF@5@5@+=feaafiart1ev1aaatCvAUfKttLearuWrP9MDH5MBPbIqV92AaeXatLxBI9gBaebbnrfifHhDYfgasaacH8akY=wiFfYdH8Gipec8Eeeu0xXdbba9frFj0=OqFfea0dXdd9vqai=hGuQ8kuc9pgc9s8qqaq=dirpe0xb9q8qiLsFr0=vr0=vr0dc8meaabaqaciaacaGaaeqabaqabeGadaaakeaaieWacuWF1bqDgaqeaaaa@2E3F@**(m/s)**	**Re**	**Nu**	h¯ MathType@MTEF@5@5@+=feaafiart1ev1aaatCvAUfKttLearuWrP9MDH5MBPbIqV92AaeXatLxBI9gBaebbnrfifHhDYfgasaacH8akY=wiFfYdH8Gipec8Eeeu0xXdbba9frFj0=OqFfea0dXdd9vqai=hGuQ8kuc9pgc9s8qqaq=dirpe0xb9q8qiLsFr0=vr0=vr0dc8meaabaqaciaacaGaaeqabaqabeGadaaakeaaieWacuWFObaAgaqeaaaa@2E25@**(W/(m**^2^**·K))**
0.0	0.000	N/A	4.47	1900
0.5	0.027	250	17.3	3400
1.0	0.053	500	24.4	4800
1.5	0.080	750	29.9	5880
2.0	0.106	1010	34.5	6790
2.5	0.133	1260	38.6	7600
3.0	0.159	1510	42.3	8320
3.5	0.186	1760	45.7	8990
4.0	0.212	2010	48.8	9610
4.5	0.239	2260	51.8	10190
5.0	0.265	2520	54.6	10740

### Calculation correction

We calculated a correction for the sensor to obtain a more accurate value of *h *since a glass layer covers the top of the Pt thin film. A thermal circuit analogous to an electric circuit was used for this correction. We treated the heat transfer-rate (*q*) as a flow. We calculated the thermal resistances from the thermal conductivity, convective heat transfer coefficient, and thickness of the material and the area of the material. The temperature difference is analogous to the potential difference. The Fourier equation [22] may be written as:

Heat flow=thermal potential differencethermal resistanceorq=ΔToverallΣRth     (11)
 MathType@MTEF@5@5@+=feaafiart1ev1aaatCvAUfKttLearuWrP9MDH5MBPbIqV92AaeXatLxBI9gBaebbnrfifHhDYfgasaacH8akY=wiFfYdH8Gipec8Eeeu0xXdbba9frFj0=OqFfea0dXdd9vqai=hGuQ8kuc9pgc9s8qqaq=dirpe0xb9q8qiLsFr0=vr0=vr0dc8meaabaqaciaacaGaaeqabaqabeGadaaakeaafaqabeqadaaabaGaeeisaGKaeeyzauMaeeyyaeMaeeiDaqNaeeiiaaIaeeOzayMaeeiBaWMaee4Ba8Maee4DaCNaeyypa0ZaaSaaaeaacqqG0baDcqqGObaAcqqGLbqzcqqGYbGCcqqGTbqBcqqGHbqycqqGSbaBcqqGGaaicqqGWbaCcqqGVbWBcqqG0baDcqqGLbqzcqqGUbGBcqqG0baDcqqGPbqAcqqGHbqycqqGSbaBcqqGGaaicqqGKbazcqqGPbqAcqqGMbGzcqqGMbGzcqqGLbqzcqqGYbGCcqqGLbqzcqqGUbGBcqqGJbWycqqGLbqzaeaacqqG0baDcqqGObaAcqqGLbqzcqqGYbGCcqqGTbqBcqqGHbqycqqGSbaBcqqGGaaicqqGYbGCcqqGLbqzcqqGZbWCcqqGPbqAcqqGZbWCcqqG0baDcqqGHbqycqqGUbGBcqqGJbWycqqGLbqzaaaabaGaee4Ba8MaeeOCaihabaGaemyCaeNaeyypa0ZaaSaaaeaacqqHuoarcqWGubavdaWgaaWcbaGaee4Ba8MaeeODayNaeeyzauMaeeOCaiNaeeyyaeMaeeiBaWMaeeiBaWgabeaaaOqaaiabfo6atjabdkfasnaaBaaaleaacqqG0baDcqqGObaAaeqaaaaaaaGccaWLjaGaaCzcamaabmaabaGaeGymaeJaeGymaedacaGLOaGaayzkaaaaaa@9225@

**Figure 6:**
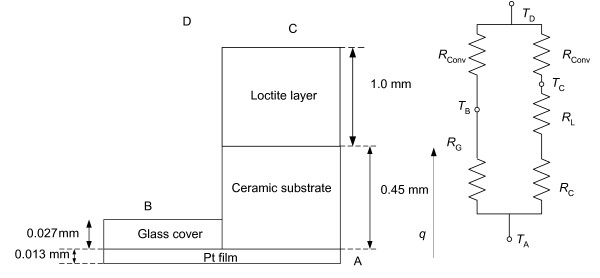
One-dimensional heat transfer through the catheter sensor, (a) the equivalent structure of the catheter sensor, (b) the electrical analogy

When the sensor is heated, the heat from the Pt thin film conducts through this thin layer of glass, which is on the top of the Pt film. The equivalent thermal resistance of the glass layer is *R*_G_. The equivalent thermal resistance of the ceramic layer is *R*_C _and the equivalent thermal resistance of the Loctite layer is *R*_L_. Convection occurs on the surface of this layer, and is included in this thermal circuit as *R*_conv_. Fig. 6 shows the one-dimensional structure of the catheter sensor and the equivalent circuit of this system. The value of *R*_G_, *R*_C_, *R*_L _and *R*_conv _can be calculated using:

RG=dGkGA     (12)
 MathType@MTEF@5@5@+=feaafiart1ev1aaatCvAUfKttLearuWrP9MDH5MBPbIqV92AaeXatLxBI9gBaebbnrfifHhDYfgasaacH8akY=wiFfYdH8Gipec8Eeeu0xXdbba9frFj0=OqFfea0dXdd9vqai=hGuQ8kuc9pgc9s8qqaq=dirpe0xb9q8qiLsFr0=vr0=vr0dc8meaabaqaciaacaGaaeqabaqabeGadaaakeaacqWGsbGudaWgaaWcbaGaee4raCeabeaakiabg2da9maalaaabaGaemizaq2aaSbaaSqaaiabbEeahbqabaaakeaacqWGRbWAdaWgaaWcbaGaee4raCeabeaakiabdgeabbaacaWLjaGaaCzcamaabmaabaGaeGymaeJaeGOmaidacaGLOaGaayzkaaaaaa@3B3A@

RC=dGkCA     (13)
 MathType@MTEF@5@5@+=feaafiart1ev1aaatCvAUfKttLearuWrP9MDH5MBPbIqV92AaeXatLxBI9gBaebbnrfifHhDYfgasaacH8akY=wiFfYdH8Gipec8Eeeu0xXdbba9frFj0=OqFfea0dXdd9vqai=hGuQ8kuc9pgc9s8qqaq=dirpe0xb9q8qiLsFr0=vr0=vr0dc8meaabaqaciaacaGaaeqabaqabeGadaaakeaacqWGsbGudaWgaaWcbaGaee4qameabeaakiabg2da9maalaaabaGaemizaq2aaSbaaSqaaiabbEeahbqabaaakeaacqWGRbWAdaWgaaWcbaGaee4qameabeaakiabdgeabbaacaWLjaGaaCzcamaabmaabaGaeGymaeJaeG4mamdacaGLOaGaayzkaaaaaa@3B2C@

RL=dLkLA     (14)
 MathType@MTEF@5@5@+=feaafiart1ev1aaatCvAUfKttLearuWrP9MDH5MBPbIqV92AaeXatLxBI9gBaebbnrfifHhDYfgasaacH8akY=wiFfYdH8Gipec8Eeeu0xXdbba9frFj0=OqFfea0dXdd9vqai=hGuQ8kuc9pgc9s8qqaq=dirpe0xb9q8qiLsFr0=vr0=vr0dc8meaabaqaciaacaGaaeqabaqabeGadaaakeaacqWGsbGudaWgaaWcbaGaeeitaWeabeaakiabg2da9maalaaabaGaemizaq2aaSbaaSqaaiabbYeambqabaaakeaacqWGRbWAdaWgaaWcbaGaeeitaWeabeaakiabdgeabbaacaWLjaGaaCzcamaabmaabaGaeGymaeJaeGinaqdacaGLOaGaayzkaaaaaa@3B5C@

and Rconv=1hconvA     (15)
 MathType@MTEF@5@5@+=feaafiart1ev1aaatCvAUfKttLearuWrP9MDH5MBPbIqV92AaeXatLxBI9gBaebbnrfifHhDYfgasaacH8akY=wiFfYdH8Gipec8Eeeu0xXdbba9frFj0=OqFfea0dXdd9vqai=hGuQ8kuc9pgc9s8qqaq=dirpe0xb9q8qiLsFr0=vr0=vr0dc8meaabaqaciaacaGaaeqabaqabeGadaaakeaacqWGsbGudaWgaaWcbaGaee4yamMaee4Ba8MaeeOBa4MaeeODayhabeaakiabg2da9maalaaabaGaeGymaedabaGaemiAaG2aaSbaaSqaaiabbogaJjabb+gaVjabb6gaUjabbAha2bqabaGccqWGbbqqaaGaaCzcaiaaxMaadaqadaqaaiabigdaXiabiwda1aGaayjkaiaawMcaaaaa@4274@

where: *d*_G _is the thickness of the glass layer = 0.027 mm, *k*_G _is the thermal conductivity of the glass = 1.38 W/(m·K), *d*_C _is the thickness of the ceramic substrate = 0.45 mm, *k*_C _is the thermal conductivity of the ceramic = 6.06 W/(m·K), *d*_L _is the thickness of the Loctite = 1.00 mm, *k*_L _is the thermal conductivity of the Loctite = 0.55 W/(m·K), *A *is the surface area of the glass layer = the surface area of the ceramic substrate = 3.2 mm × 1.9 mm = 6.08 × 10^-6^ m^2^

Heat from the layer of thin film Pt also conducts through the ceramic substrate and Loctite layer as shown in Fig. 6. The combination of the thermal resistance of the Loctite layer (*R*_L_) and the thermal resistance of the ceramic substrate (*R*_C_) is parallel to the thermal resistance of the glass layer. Thus for the catheter sensor, we calculate *h*, using:

h=1A[[ΔTq−RG+RL+RC2]+(RG+RL+RC)24+(ΔTq)2−RG(RL+RC)]     (16)
 MathType@MTEF@5@5@+=feaafiart1ev1aaatCvAUfKttLearuWrP9MDH5MBPbIqV92AaeXatLxBI9gBaebbnrfifHhDYfgasaacH8akY=wiFfYdH8Gipec8Eeeu0xXdbba9frFj0=OqFfea0dXdd9vqai=hGuQ8kuc9pgc9s8qqaq=dirpe0xb9q8qiLsFr0=vr0=vr0dc8meaabaqaciaacaGaaeqabaqabeGadaaakeaacqWGObaAcqGH9aqpdaWcaaqaaiabigdaXaqaaiabdgeabnaadmaabaWaamWaaeaadaWcaaqaaiabfs5aejabdsfaubqaaiabdghaXbaacqGHsisldaWcaaqaaiabdkfasnaaBaaaleaacqqGhbWraeqaaOGaey4kaSIaemOuai1aaSbaaSqaaiabbYeambqabaGccqGHRaWkcqWGsbGudaWgaaWcbaGaee4qameabeaaaOqaaiabikdaYaaaaiaawUfacaGLDbaacqGHRaWkdaGcaaqaamaalaaabaGaeiikaGIaemOuai1aaSbaaSqaaiabbEeahbqabaGccqGHRaWkcqWGsbGudaWgaaWcbaGaeeitaWeabeaakiabgUcaRiabdkfasnaaBaaaleaacqqGdbWqaeqaaOGaeiykaKYaaWbaaSqabeaacqaIYaGmaaaakeaacqaI0aanaaGaey4kaSYaaeWaaeaadaWcaaqaaiabfs5aejabdsfaubqaaiabdghaXbaaaiaawIcacaGLPaaadaahaaWcbeqaaiabikdaYaaakiabgkHiTiabdkfasnaaBaaaleaacqqGhbWraeqaaOGaeiikaGIaemOuai1aaSbaaSqaaiabbYeambqabaGccqGHRaWkcqWGsbGudaWgaaWcbaGaee4qameabeaakiabcMcaPaWcbeaaaOGaay5waiaaw2faaaaacaWLjaGaaCzcamaabmaabaGaeGymaeJaeGOnaydacaGLOaGaayzkaaaaaa@6987@

where *A *is the exposed surface area of the catheter sensor = 1.9 mm × 3.2 mm

### In vitro experimental results

**Figure 7:**
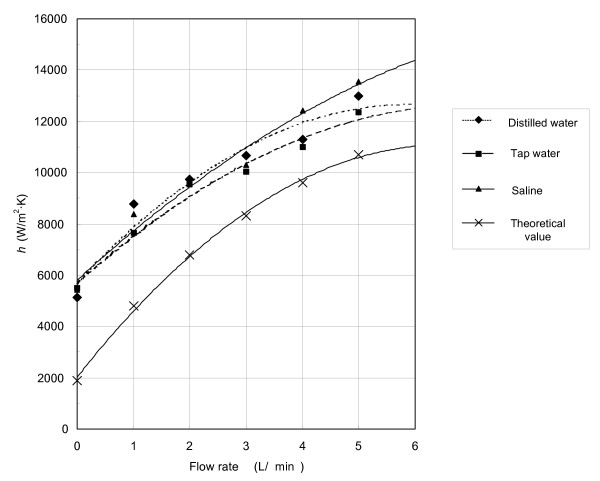
In vitro experimental results of *h *at various flow rates in distilled water, pure water and saline performed at 39°C.

Fig. 7 shows the value of *h *from 16 experiments (for each flow rate) at various flow rates in three different types of media: distilled water, tap water and saline. Table 4 shows the average values of *h *and the standard deviations of the values of *h *from each flow rate. All of the *in vitro *experiments were performed at 39°C similar to the swine body temperature.

**Table 4:** The averages and standard deviations of *h *at different flow rates

**Flow (L/min)**	**Distilled water (W/(m^2^·K))**		**Tap water (W/(m^2^·K))**		**Saline (W/(m^2^·K))**	
	
	Average	SD	Average	SD	Average	SD
0.0	5100	500	5500	400	5400	400
1.0	9000	1200	8000	1100	8400	800
2.0	10100	1800	9500	1100	9700	1100
3.0	12000	1800	10300	800	10300	1400
4.0	12200	1900	11000	400	12400	1800
5.0	13000	1800	12300	800	13600	1700

The experimental results show that from 0 to 5 L/min, values of *h *in W/(m^2^·K) were for distilled water 5100 to 13000, for tap water 5500 to 12300, and for saline 5400 to 13600 (as shown in Fig. 7). At low flow rates (from 1 to 3 L/min), distilled water yielded the highest value of *h *(8700 W/(m^2^·K) at 1 L/min, which was about 13% higher than the lowest value of *h *obtained from tap water). At the higher flow rates (from 4 L/min or higher), saline yielded the highest value of *h *(13600 W/(m^2^·K) at 5 L/min, which was about 9% higher than the lowest value of *h *obtained from tap water). No significant differences of *h *among these three media were found at any flow rate.

It is important to consider the closest theoretical prediction possible in order to guide our experimental testing. Therefore, we compared the experimental results with the theoretical values and found that at all flow rates, the *h *results from the *in vitro *experiments of those three types of media yielded a higher value of approximately 1500 W/(m^2^·K) above the theoretical value.

## Discussion

We have built an instrument for the measurement of *h *and tested it in distilled water, tap water and saline. The measured *h *varied significantly with flow rate, as theory suggests it should, but did not follow the square root dependence on Reynolds number that was expected. The present data varied approximately with the 0.3 power, whereas theory suggests 0.5.

Because of the small size of the sensor, the Grashof or Rayleigh Number and the Reynolds number (for the forced convection flows) were small. Most of the correlations were more reliable at the upper end of the Grashof or Reynolds number ranges, hence the experimental data agreed better with the theoretical calculation at higher flows.

At the lower flow rate in stagnant liquid (at low Grashof Number), the experimental flow regime is uncertain. The natural convection correlation assumes a flat vertical surface, no edge effects, constant fluid properties, and no forced convection (fluid movement) at all, which are difficult to replicate. Most other experiments in natural convection attempt to achieve relatively large Grashof numbers, where the flow is strongly driven by free convection with negligible small amounts of forced convection. However, in our case, Gr was small, so normally negligible small forced convection had a big effect and could not be neglected.

The data changed slightly with the type of medium. All three data sets were within ± 7% of the mean line (excluding the free convection results, which were not expected to lie on the same lines as forced convection). No leakage current was detected in all *in vitro *experiments, which suggested that this system should be safe to perform the measurement *in vivo*.

We did not measure *h *at the tube wall *in vitro *because the tube wall was rigid and the sensor would have been forced away from the wall into flow streamlines different from those at the tube wall. This is the reason we measured *h *on the axis, with known flow. Once we calibrated *h *on the axis, we later used the sensor *in vivo *against the soft tissue of the endocardium or vessels to measure *h*. The sensor was small enough so that it indented the soft tissue to place the sensor face close to the wall contours and achieve minimal disruption of the flow streamlines at the endocardium or vessel wall. This enabled measurement of the value of *h *at the endocardium or vessel wall, which was less than the value of *h *away from the wall.

## Conclusion

We believe this system is the smallest, most accurate method of minimally invasive measurement of *in vivo h *in animals and provides the least disturbance of flow.

## Competing interests

The author(s) declare that they have no competing interests.

## Authors' contributions

CT carried out the design of the test equipment, ran tests, and drafted the manuscript. LC ran tests. JGW conceived of the study, and participated in its design and coordination and helped to draft the manuscript. PVF contributed to the heat transfer sections. All authors read and approved the final manuscript.
